# Handgrip Strength and Dehydroepiandrosterone Sulfate in a Frailty Unit: A Retrospective Study

**DOI:** 10.7759/cureus.69753

**Published:** 2024-09-19

**Authors:** Daniano Caires, Miguel Homem Costa, João Miguel Freitas, Rafael Ferreira Nascimento, Tiago Teófilo, Luís Ramos dos Santos, João Gouveia, Carolina Carvalhinha

**Affiliations:** 1 Physical Medicine and Rehabilitation, Hospital Central do Funchal, Funchal, PRT; 2 Internal Medicine, Hospital Central do Funchal, Funchal, PRT

**Keywords:** dehydroepiandrosterone sulfate, frail scale, frailty, frailty unit, handgrip strength

## Abstract

Introduction: Frailty is characterized by vulnerability and decline in physical, mental, and social activity, significantly contributing to adverse health outcomes. Frailty encompasses nutritional status, muscle strength, inflammation, and hormones. Dehydroepiandrosterone sulfate (DHEAS) is one of the hormones hypothesized to play a role in frailty. Handgrip strength (HGS) correlates with overall muscle strength. The fatigue, resistance, ambulation, illnesses, and loss of weight (FRAIL) scale can be used to readily screen frailty. Identifying markers associated with frailty can facilitate its early diagnosis, risk stratification, and target interventions to prevent or mitigate its negative consequences. This study sought to evaluate the associations between frailty, HGS, and DHEAS in a Portuguese frailty unit (FU).

Methods: We developed an observational retrospective study in an FU. Patients admitted to the FU underwent a rehabilitation program. We assessed frailty with the FRAIL scale. We assayed DHEAS upon admission to the FU. We measured HGS at admission (i-HGS) and discharge (f-HGS). We also considered HGS variation (∆ HGS) and length of stay.

Results: Out of 119 subjects, 97 fulfilled the eligibility criteria (mean age 78.35 ± 9.58 years; 44.33% men). Overall, 88 (90.72%) patients had a FRAIL scale score of 3 or more. DHEAS values were not significantly different in either the categories of the FRAIL scale or frailty status. DHEAS values were also not significantly correlated with either i-HGS, f-HGS, ∆ HGS, age, or FU length of stay. Frail patients had a significantly lower i-HGS (p = 0.002) and f-HGS (p = 0.001) and a significantly higher length of stay (p = 0.006). Also, the i-HGS and f-HGS significantly decreased with the increase of the FRAIL scale score (p < 0.0001 for both). The cut-off values of the i-HGS and the f-HGS for detecting frail patients in our study were 13.3 kg and 19.1 kg, respectively (p < 0.0001 for both). The i-HGS was significantly and independently associated with the frailty status of frail (p = 0.001), with a 15% probability reduction of a patient being frail for every kilogram increase in the i-HGS.

Conclusion: Frail patients assessed with the FRAIL scale had a significantly lower i-HGS and f-HGS and a higher length of stay. In this study, we found frailty and DHEAS to be not associated and DHEAS values to be not correlated with i-HGS or f-HGS. In our opinion, the creation of an FU with an initial FRAIL scale screening and HGS measurement might have a significant impact on identifying frail people and ensuring the implementation of a multimodal multidisciplinary approach.

## Introduction

Frailty is a clinical syndrome characterized by vulnerability and decline in physical, mental, and social activity, being more common as we age but not exclusive to the older adult population [1-4]. It is worth noting that frailty is a complex multifactorial condition influenced by nutrition, overall muscle strength, and hormones [2].

In 2008, the International Academy of Nutrition, Health, and Aging proposed the fatigue, resistance, ambulation, illnesses, and loss of weight (FRAIL) scale, a simple, feasible, and practical screening test to identify prefrail and frail people [1,2,5]. This scale assesses five domains: fatigue, resistance, ambulation, illnesses, and loss of weight [1,2]. Ever since it was introduced, this tool has undergone validation across populations and has seen increasing use in both clinical and research settings [5,6-11].

Dehydroepiandrosterone sulfate (DHEAS) is a steroid hormone for which the adrenal glands are the sole source in women and the principal source in men. It is an important precursor of androgen and estrogen biosynthesis. DHEAS reaches peak levels in young adulthood and declines with aging [12,13]. The decline in DHEAS levels has often been implicated in age-related physiological changes, comprising overall muscle strength reduction and decreased physical activity as well as the development of various age-related conditions, including frailty [12,14-18].

Handgrip strength (HGS) is a widely recognized indicator of overall muscle strength [19,20] and it correlates with various health outcomes, including disability, functional status, hospital length of stay, morbidity, and mortality [21,22]. A multifactorial and interdisciplinary intervention can be used to address frailty [23]. There is growing evidence to suggest that exercise, particularly resistance and aerobic training, can play a pivotal role in preventing and managing frailty [23-31]. Furthermore, a personalized nutritional intervention, placing particular emphasis on protein intake, hydration, and caloric balance, can positively affect various aspects of frailty [27,32,33]. In addition, screening tools for older persons' prescriptions (STOPP) and screening tools to alert them to the right treatment (START) criteria should be used to optimize medication management, especially in the context of frailty and old age [34-37]. Therefore, to guarantee this comprehensive and optimized multimodal multidisciplinary approach, the creation of a frailty unit (FU) is recommended to deliver high-quality, sustainable, and cost-effective health care [38-40]. The aim of this study was to determine the associations amongst frailty, HGS, and DHEAS in a Portuguese FU.

## Materials and methods

Study design and participants

This longitudinal study was conducted at the FU of the Autonomous Region of Madeira Health Care System (SESARAM) between July 15, 2021 and August 31, 2022. We reviewed the demographic, laboratory, and clinical data from 119 adult patients admitted to the FU. Patients came from the internal medicine inpatient ward and were considered for admission at the FU once the main cause of hospitalization was resolved or managed. Exclusion criteria include pregnant women; non-frail; deaths occurring during admission; use of estrogen, DHEAS, or androgen in the past six months; upper limb paralysis; upper limb amputations; and mentally impaired patients. The ethics committee of SESARAM approved the current study (nº38/2022) by the Helsinki Declaration statements, and the requirement for written informed consent was waived.

Frailty assessment

We assessed frailty using the FRAIL scale. This screening tool ranged from 0 to 5 points and comprised five domains: fatigue, resistance, ambulation, illnesses, and loss of weight [1,2]. We scored each question 1 point if answered “yes” and 0 points if answered “no.” We measured fatigue by asking patients if they felt tired most of the time during the previous four weeks. We assessed resistance by asking if they had any difficulty walking up 10 steps alone without resting and without aids. We determined ambulation by asking whether they had any difficulty walking several hundred meters alone and without aids. We evaluated illness by asking if they had 5 or more illnesses out of the following: hypertension, diabetes mellitus, cancer (other than minor skin cancer), chronic lung disease, myocardial infarction, angina, congestive heart failure, cerebral vascular disease, asthma, arthritis, and kidney disease. We analyzed loss of weight by asking if respondents had a weight decline of more than 5% within the past 12 months [1,2].

We then obtained frailty status by summing the total number of points. We considered patients with a total of 0 points on the FRAIL scale as robust, those with 1 or 2 points as prefrail, and those with 3 or more points as frail [1,2].

Laboratory tests

We obtained serum levels of DHEAS at patients’ admission. We measured DHEAS using electrochemiluminescence immunoassay Elecsys (F. Hoffmann-La Roche Ltd., Basel, Switzerland).

Handgrip strength protocol

We measured HGS at admission (i-HGS) and discharge (f-HGS) of the FU with a dynamometer KERN MAP 80K1 (KERN & SOHN GmbH, Balingen, Germany) to the nearest 0.01 kg. We also calculated and considered the HGS variation (∆ HGS). We asked patients about their dominant hand, and if they could not provide a straightforward answer, we further questioned them about the hand they perceived to be stronger and/or which hand they would use to hold the spoon if eating soup.

Patients were seated in an upright posture, with arms by their sides, elbows flexed to 90°, and forearms in a neutral position. We instructed them to use their dominant hand to squeeze the dynamometer as hard as they could for five seconds. They made two attempts, one minute apart, to avoid fatigue. The first one was a trial to familiarize the patient with the technique, and we used the second one to assess HGS.

Rehabilitation program

Patients underwent a personalized moderate-intensity multi-component exercise intervention consisting of at least 45-60 minutes of progressive resistance and aerobic exercise five times per week. The aerobic exercise consisted of indoor cycling, pedal exerciser, or treadmill walking. The progressive resistance program involved all major muscle groups of the upper and lower extremities and trunk. Moderate-intensity exercise was achieved when patients perceived increased sweating and breathing became heavier, but patients could still hold short conversations. Studies have demonstrated that moderate-intensity multi-component training designed for improving muscle strength and cardiorespiratory endurance is an effective method for enhancing functional performance in older adults [24,41-46].

We provided participants with customized caloric and protein support, including daily supplementation with 3 g of calcium β-hydroxy-β-methylbutyrate (Ca-HMB). The PROT-AGE study group and the European Society for Clinical Nutrition and Metabolism have proposed that older adults should consume between 1.0-1.5 g/kg/day of protein [33,47]. Furthermore, the leucine catabolite Ca-HMB has been shown to independently stimulate muscle protein synthesis [48,49] and, when combined with resistance exercises, resulted in greater fat loss and greater muscle strength gains in older adults [50,51].

We obtained a reduction of polypharmacy using the “STOPP” and “START” criteria [35-37]. The intervention plan also included the management of intercurrent illnesses and comorbidities. We provided health literacy to all patients, educating them about fall prevention, diet, and physical exercise.

Other items of interest

We also considered age, gender, and length of stay at the FU.

Statistical approach

We present the data as frequency and respective percentage for categorical variables. Continuous variables are expressed as mean ± standard deviation or median (minimum-maximum). We used the Kolmogorov-Smirnov test to assess the normality of the continuous variables. For non-normally distributed data we used the non-parametric Mann-Whitney U test to assess the difference between mean values of dichotomous variables and the ANOVA test for the polychotomous variables. We applied post-hoc tests. We assessed the homogeneity of variances with Levene’s test. We used Spearman’s correlation test for correlation analysis. We used the Jonckheere-Terpstra test to determine the significance of a trend. We applied multivariate logistic regression analysis using the forward Wald method to identify variables independently associated with frail patients. We used receiver operating characteristics (ROC) curve analysis to determine the cut-off values of continuous parameters and to determine the area under the curve (AUC). We performed data analysis using IBM SPSS Statistics for Windows version 25 (IBM Corp., Armonk, NY, USA), and we considered a p-value < 0.05 as statistically significant.

## Results

During the study period, 119 patients were admitted to the FU. Of those, eight (6.72%) died after admission. We also excluded three (2.52%) patients with upper limb paralysis, one (0.84%) patient with the use of androgen, and 10 (8.40%) patients with missing data. We then included a total of 97 adult patients in this study (43 men and 54 women; mean age 78.35 ± 9.58 years).

Female patients were older than male patients (80.20 ± 8.24 vs 76.02 ± 10.68). A FRAIL scale of 4 points was the most common score (n = 52, 53.61%) for both male (n = 24, 55.81%) and female patients (n = 28, 51.85%). We cataloged most patients with a frailty status of frail (n = 88, 90.72%). The baseline characteristics of the study population are presented in Table 1.

**Table 1:** Baseline characteristics of the study population.

	All (n = 97)	Male (n = 43)	Female (n = 54)
Age, years	78.35 ± 9.58	76.02 ± 10.68	80.20 ± 8.24
FRAIL scale, n (%)			
1	3 (3.09)	2 (4.65)	1 (1.85)
2	6 (6.19)	4 (9.30)	2 (3.70)
3	33 (34.02)	13 (30.23)	20 (37.04)
4	52 (53.61)	24 (55.81)	28 (51.85)
5	3 (3.09)	0 (0.00)	3 (5.56)
Frailty status, n (%)			
Prefrail	9 (9.28)	6 (13.95)	3 (5.56)
Frail	88 (90.72)	37 (86.05)	51 (94.44)

The mean DHEAS level was 53.37 ± 51.63 µg/dL, with men having a significantly higher value (69.98 ± 67.45 µg/dL vs 40.14 ± 28.67 µg/dL, p = 0.012). The mean i-HGS was 13.03 ± 6.85 kg and the f-HGS was 16.36 ± 7.11 kg with male patients having a significantly higher value in both i-HGS (16.88 ± 7.80 kg vs 9.96 ± 3.90 kg, p < 0.0001) and f-HGS (20.64 ± 7.75 kg vs 12.96 ± 4.17 kg, p < 0.0001). The mean ∆ HGS was 3.34 ± 3.49 kg. The mean length of stay at the FU was 21.71 ± 16.48 days. There were no significant differences between male and female patients regarding ∆ HGS (p = 0.41) and length of stay (p = 0.896). Table 2 shows the laboratory and clinical characteristics of the study subjects.

**Table 2:** Laboratory and clinical characteristics of the study population. DHEAS: dehydroepiandrosterone sulfate; i-HGS: initial handgrip strength; f-HGS: final handgrip strength; ∆ HGS: handgrip strength variation; FU: frailty unit; p < 0.05 considered statistically significant.

	All (n = 97)	Male (n = 43)	Female (n = 54)	P-value
DHEAS, µg/dL	53.37 ± 51.63	69.98 ± 67.45	40.14 ± 28.67	0.012
i-HGS, kg	13.03 ± 6.85	16.88 ± 7.80	9.96 ± 3.90	< 0.0001
f-HGS, kg	16.36 ± 7.11	20.64 ± 7.75	12.96 ± 4.17	< 0.0001
∆ HGS, kg	3.34 ± 3.49	3.76 ± 4.43	3.00 ± 2.49	0.410
FU length of stay, days	21.71 ± 16.48	22.95 ± 18.98	20.72 ± 14.29	0.896

We ran a Spearman’s correlation test to determine the relationship between the DHEAS values and HGS, age, and FU length of stay (Table 3). There was a non-significant weak correlation between the DHEAS and the i-HGS, f-HGS, and FU length of stay (rho = 0.039, p = 0.703; rho = 0.082, p = 0.424; rho = 0.017, p = 0.870, respectively). We observed a non-significant very weak negative correlation between DHEAS and ∆ HGS and age (rho = -0.072, p = 0.485; rho = -0.134, p = 0.190, respectively).

**Table 3:** Correlation analysis of DHEAS values with HGS, age, and FU length of stay. DHEAS: dehydroepiandrosterone sulfate; i-HGS: initial handgrip strength; f-HGS: final handgrip strength; ∆ HGS: handgrip strength variation; FU: frailty unit; Rho: spearman’s correlation coefficient; p < 0.05 considered statistically significant.

		N	Rho	P-value
DHEAS	i-HGS, kg	97	0.039	0.703
	f-HGS, kg	97	0.082	0.424
	∆ HGS, kg	97	-0.072	0.485
	Age, years	97	-0.134	0.190
	FU length of stay, days	97	0.017	0.870

We also performed the same correlation analysis separately for male and female patients (data not shown). In male patients, there was no significant correlation between DHEAS and the i-HGS, f-HGS, and ∆ HGS (p = 0.482, p = 0.462, p = 0.129, respectively). The same was observed in female patients (p = 0.730, p = 0.872, p = 0.735, respectively).

Furthermore, when considering the expected values of DHEAS using Elecsys for gender and age as listed in the method sheet, only 10 frail male patients (10.31%) were out of the expected DHEAS 5-95th percentile, with seven of those (7.22%) being below that percentile (data not shown).

A comparison of demographic, laboratory, and clinical characteristics between prefrail and frail is shown in Table 4. Compared with prefrail patients, frail patients had a significantly lower i-HGS (12.18 ± 6.21 kg vs 21.29 ± 7.69 kg, p = 0.002) and f-HGS (15.57 ± 6.82 kg vs 24.08 ± 5.21 kg, p = 0.001) and a significantly higher length of stay (22.88 ± 16.78 days vs 10.33 ± 6.00 days, p = 0.006). There were no significant differences between prefrail and frail patients regarding age, sex, DHEAS levels, and ∆ HGS.

**Table 4:** Comparison of clinical and demographic characteristics between prefrail and frail patients. DHEAS: dehydroepiandrosterone sulfate; i-HGS: initial handgrip strength; f-HGS: final handgrip strength; ∆ HGS: handgrip strength variation; FU: frailty unit; p < 0.05 considered statistically significant.

	Prefrail (n = 9)	Frail (n = 88)	P-value
Age, years	74.33 ±8 .79	78.76 ± 9.61	0.188
Male, n (%)	6 (66.67%)	37 (42.05%)	0.179
DHEAS, µg/dL	57.83 ± 45.09	52.91 ± 52.47	0.593
i-HGS, kg	21.29 ± 7.69	12.18 ± 6.21	0.002
f-HGS, kg	24.08 ± 5.21	15.57 ± 6.82	0.001
∆ HGS, kg	2.79 ± 2.74	3.39 ± 3.56	0.584
FU length of stay, days	10.33 ± 6.00	22.88 ± 16.78	0.006

Table 5 shows the clinical characteristics stratified by the FRAIL scale score. The i-HGS and f-HGS were significantly different based on the different categories of the FRAIL scale, both decreasing with an increase in the FRAIL scale score (p < 0.0001). There were no significant differences between the different categories of the FRAIL scale and the DHEAS levels (p = 0.811), ∆ HGS (p = 0.198), and length of stay (p = 0.165).

**Table 5:** Comparison of clinical characteristics in the FRAIL scale. DHEAS: dehydroepiandrosterone sulfate; i-HGS: initial handgrip strength; f-HGS: final handgrip strength; ∆ HGS: handgrip strength variation; FU: frailty unit; FRAIL: fatigue, resistance, ambulation, illnesses, and loss of weight; p < 0.05 considered statistically significant.

	FRAIL scale 1 (n = 3)	FRAIL scale 2 (n = 6)	FRAIL scale 3 (n = 33)	FRAIL scale 4 (n = 52)	FRAIL scale 5 (n = 3)	P-value
DHEAS, µg/dL	78.87 ± 59.66	47.32 ± 37.83	48.78 ± 28.91	56.53 ± 63.98	35.63 ± 30.35	0.811
i-HGS, kg	22.10 ± 6.56	20.88 ± 8.76	12.52 ± 6.73	12.45 ± 5.75	3.83 ± 1.96	< 0.0001
f-HGS, kg	24.33 ± 4.17	23.95 ± 6.04	17.01 ± 7.73	15.26 ± 5.80	5.20 ± 3.58	< 0.0001
∆ HGS, kg	2.23 ± 2.39	3.07 ± 3.07	4.48 ± 4.30	2.82 ± 2.93	1.37 ± 1.63	0.198
FU length of stay, days	10.00 ± 3.00	10.50 ± 7.34	21.33 ± 15.26	24.40 ± 17.92	13.33 ± 10.12	0.165

In the multivariate logistic regression analysis of frail patients (Table 6), the only variable that remained in the model was i-HGS, which was significantly and independently associated with a frailty status of frail (p = 0.001), indicating that for every kilogram increase in the i-HGS, the probability of a patient being frail decreased by 15%.

**Table 6:** Logistic regression analysis of frail patients and confounding variables. Forward Wald method. Age, sex, dehydroepiandrosterone sulfate and frailty unit length of stay left the equation. CI: confidence interval; df: degrees of freedom; i-HGS: initial handgrip strength; OR: odds ratio; SE: standard error; p < 0.05 considered statistically significant.

	Beta	SE	Wald	df	OR (CI 95%)	P-value
i-HGS	-0.158	0.048	10.860	1	0.854 (0.777 - 0.938)	0.001
Constant	4.855	1.003	23.442	1	128.354	< 0.0001

We conducted ROC analysis for the variables and identified the threshold for discriminating the prefrail and frail patients (Table 7), which was 13.3 kg for i-HGS (AUC 0.820, p < 0.0001), 19.1 kg for f-HGS (AUC 0.845, p < 0.0001) and 13 days for FU length of stay (AUC 0.780, p < 0.0001).

**Table 7:** ROC analysis of i-HGS, f-HGS and FU length of stay in frail patients. AUC: area under the curve; CI: confidence interval; i-HGS: initial handgrip strength; f-HGS: final handgrip strength; FU: frailty unit; p < 0.05 considered statistically significant.

	Cut-off values	AUC (95% CI)	P-value
i-HGS, kg	13.3	0.820	< 0.0001
f-HGS, kg	19.1	0.845	< 0.0001
FU length of stay, days	13.0	0.780	< 0.0001

## Discussion

This study aimed to determine the associations between frailty, HGS, and DHEAS in a Portuguese FU. In our study, 88 subjects (90.72%) had a FRAIL scale score of 3 or more points, thus being categorized as frail.

As expected from the literature, DHEAS was significantly higher in male patients [13,52], as were also the i-HGS and f-HGS [53]. Interestingly, the DHEAS values were not significantly correlated with either i-HGS, f-HGS, ∆ HGS, age, or FU length of stay. In a younger and middle-aged population, DHEAS values were positively correlated with better performance on tests of physical functioning; however, in older adults, as in this study, these associations were often inconsistent [15,54].

Moreover, DHEAS was not significantly different in either the categories of the FRAIL scale or based on frailty status. Previous research has shown that DHEAS has a higher impact on frailty for levels extremely below the expected range, rather than just within the expected range’s lower limit. Such low levels are associated with more chronic illness conditions [15] and linked to higher cardiovascular disease mortality and all-cause mortality [55,56]. In our study, only seven frail patients (7.22%) were below the 5-95th percentile for age and gender, which was a limited number that hindered the ability to draw conclusive findings.

Frail patients had a significantly lower i-HGS and f-HGS and a significantly higher length of stay. Also, the i-HGS and f-HGS significantly decreased with the increase of the FRAIL scale score. The cut-off values of the i-HGS and the f-HGS for detecting frail patients in our study were 13.3 Kg and 19.1 Kg, respectively. The usefulness of these cut-off values requires further investigation in a larger-scale clinical study. The i-HGS was significantly and independently associated with frailty status of frail, in which for every kilogram increase in the i-HGS there was a 15% reduction in the likelihood of a patient being frail. These findings are in consonance with the literature, where HGS may be especially useful in the context of frailty, disability, and multimorbidity, identifying patients at high risk of adverse outcomes [19-22,57-59]. Furthermore, in a study encompassing more than 140,000 participants, HGS was a better predictor of all-cause and cardiovascular mortality than systolic blood pressure [60].

The FRAIL scale is a simple and straightforward screening test that, despite not being validated for the Portuguese population, may be used to promptly identify frail people, enabling a more in-depth assessment of those patients [5-10,61]. We advocate for the HGS measurement both initially in conjunction with the application of the FRAIL scale and at the end of the FU stay because it constitutes a valuable diagnostic component of frailty, and it is a reliable indicator of overall muscle strength [21,62]. It also correlates with diverse health outcomes, including disability, functional status, hospital length of stay, morbidity, and mortality [21,22].

Our study had several limitations. First, the sample size of 97 was small, with only nine patients being prefrail (9.28%), of whom only three were female (5.56%). Second, HGS was not adjusted for sex and age; however, a meta-analysis by Cooper, et al. demonstrated that sex difference in HGS diminishes with increasing age [63]. Third, we should consider that despite encouragement, HGS can be influenced by the motivation of the subject during the assessment. Fourth, the FRAIL scale relies on self-reporting, and some answers might have been affected by unrecognized cognitive impairment. Furthermore, the FRAIL scale has yet to be validated in the Portuguese population. Fifth, this study is constrained by inherent selection bias because of retrospective data collection.

## Conclusions

Frail patients assessed using the FRAIL scale had a significantly lower i-HGS and f-HGS and higher length of stay. The thresholds for the i-HGS and the f-HGS for detecting frail patients were 13.3 kg and 19.1 kg, respectively. An additional kilogram in the i-HGS reduced the likelihood of being frail by 15%. In this study, we found frailty and DHEAS to be not associated.

Frailty is a high-burden, manageable condition, and the creation of an FU may play a pivotal role in identifying frail people and ensuring the implementation of a thorough and efficient multimodal multidisciplinary approach. Further prospective research with a large sample size would be beneficial to validating our findings and addressing the study’s limitations.
